# Sodium First Approach, to Reset Our Mind for Improving Management of Sodium, Water, Volume and Pressure in Hemodialysis Patients, and to Reduce Cardiovascular Burden and Improve Outcomes

**DOI:** 10.3389/fneph.2022.935388

**Published:** 2022-07-07

**Authors:** Bernard Canaud, Jeroen Kooman, Andreas Maierhofer, Jochen Raimann, Jens Titze, Peter Kotanko

**Affiliations:** ^1^ School of Medicine, Montpellier University, Montpellier, France; ^2^ Global Medical Office, Freseenius Medical Care (FMC)-France, Fresnes, France; ^3^ Maastricht University Maastricht Medical Center (UMC), Maastricht University, Maastricht, Netherlands; ^4^ Global Research Development, Fresenius Medical Care (FMC) Deutschland GmbH, Bad Homburg, Germany; ^5^ Research Division, Renal Research Institute, New York, NY, United States; ^6^ Cardiovascular and Metabolic Disease Programme, Duke-National University Singapore (NUS) Medical School, Singapore, Singapore; ^7^ Nephrology, Icahn School of Medicine at Mount Sinai, New York, NY, United States

**Keywords:** chronic kidney disease stage 5D, hemodialysis, fluid overload, hypertension, cardiac disease, sodium and water imbalance

## Abstract

New physiologic findings related to sodium homeostasis and pathophysiologic associations require a new vision for sodium, fluid and blood pressure management in dialysis-dependent chronic kidney disease patients. The traditional dry weight probing approach that has prevailed for many years must be reviewed in light of these findings and enriched by availability of new tools for monitoring and handling sodium and water imbalances. A comprehensive and integrated approach is needed to improve further cardiac health in hemodialysis (HD) patients. Adequate management of sodium, water, volume and hemodynamic control of HD patients relies on a stepwise approach: the first entails assessment and monitoring of fluid status and relies on clinical judgement supported by specific tools that are online embedded in the HD machine or devices used offline; the second consists of acting on correcting fluid imbalance mainly through dialysis prescription (treatment time, active tools embedded on HD machine) but also on guidance related to diet and thirst management; the third consist of fine tuning treatment prescription to patient responses and tolerance with the support of innovative tools such as artificial intelligence and remote pervasive health trackers. It is time to come back to sodium and water imbalance as the root cause of the problem and not to act primarily on their consequences (fluid overload, hypertension) or organ damage (heart; atherosclerosis, brain). We know the problem and have the tools to assess and manage in a more precise way sodium and fluid in HD patients. We strongly call for a sodium first approach to reduce disease burden and improve cardiac health in dialysis-dependent chronic kidney disease patients.

## 1 Introduction

Despite recent improvements in dialysis patient outcomes (1–3), cardiovascular events remain the leading cause of death accounting for 50 to 55% of mortality according to estimates of the United States Renal Data System (3). Severe arrhythmias and sudden cardiac death account for almost 28% of cardiac death while coronary ischemic disease, congestive heart failure or vascular events are responsible for the rest (3).

Findings of recent studies may be taken as examples to highlight the burden of this problem. Fluid overload is quite common in hemodialysis patients. In a large cohort of incident hemodialysis patients (>40.000), moderate fluid overload (>2.5 l) assessed by multifrequency bioimpedance was noted in 46% of patients, while a more severe one (>6 l) was observed in about 10% (4, 5). Hypertension, even when set as a predialysis systolic blood pressure >160 mmHg, was noted in 20% of patients in the same cohort and more frequently associated with fluid overload (4). In addition, cardiac health issues tended to aggravate over the next 12 months in about half of the patients, contributing to worsened outcomes and almost doubling the relative risk of death (4). Left ventricular hypertrophy (LVH), a surrogate marker of chronic fluid overload and/or hypertension was detected up to 75% in patients starting dialysis with a continuous increase over time (6–8). Hyponatremia, a biomarker strongly associated with poor outcome in dialysis patients, is observed in 10 to 19% of hemodialysis patients in recent cohort reports (9, 10). In a recent study, it has been shown that hyponatremia was in fact associated with combined fluid overload (EC and IC fluid excess) with intercompartmental fluid imbalance, translating into the occurrence of an intercurrent illness (cardiac failure, inflammation, oxidative stress) being likely associated with protein energy wasting (10–12). This observation is in line with findings large cohort studies (13, 14) and confirm validity of a recent proposed workflow algorithm to explore hyponatremia in dialysis patients (15). A high prevalence of cardiac arrhythmias was shown in studies using implanted loop recorders (16–18). Recent cardiac rhythm monitoring study involving sixty-six patients using continuous monitoring of such loop recorders have identified that 1678 clinically significant arrhythmias (CSA) were observed in 44 (66.7%) dialysis patients (16, 19). The majority were bradycardia not necessarily with hyperkalemia (19.7%) followed by asystole (9.1%) and ventricular tachycardia (1.5%). Confirmed arrhythmia subtypes were represented by atrial arrhythmia (90.9%) with atrial fibrillation (30%) followed by ventricular arrhythmia (71.3%) and bradyarrhythmia (25.8%). Five patients of this cohort with serious bradyarrhythmia required pacemaker implantation to prevent cardiac sudden death. Lastly, pulmonary edema and related congestive heart decompensation episodes are among the most frequent causes of hospitalization (44%) and readmissions creating a significant burden both on patient and healthcare system (20, 21).

Fluid volume depletion and care management of hemodialysis patients is another critical point that may affect outcomes (22, 23). As documented in recent reports, too aggressive dry weight policy based on high ultrafiltration rate (24, 25) (> 13 ml/hr/kg, for example), is associated with critical hypovolemia and serious intradialytic hypotension (IDH) (26), that may lead to repetitive systemic hemodynamic stress episodes with end-organ damage (27, 28). Repetitive ischemic insults result from inadequate hemodynamic response to volume depletion but not only (24, 29). In fact, ischemic insult is part of a broader multifactorial stress condition, namely dialysis-induced systemic stress syndrome, that includes hemobiological reactions, hypoxemia, thermal imbalance, osmotic and electrolytic shifts (27). As recently summarized, dialysis-induced systemic stress may contribute to morbidity and mortality in dialysis patients as a potent disease modifier including protein energy wasting process (27, 29). A call for action is needed to mitigate this additional cardiovascular risk in maintenance hemodialysis patients (27).

In this context, it is easily recognized that sodium and water related disorders contribute significantly to cardiac burden in hemodialysis patients, either from chronic fluid overload exposure during the interdialytic period or from acute fluid depletion during dialytic time (30, 31). Now, it must be highlighted that sodium and fluid accumulation is a long-standing process aggravating along chronic kidney disease progression with a culminant point at the end stage of kidney disease. In addition, specific conditions (i.e., aging) or diseases (i.e., hypertension, diabetes) are strong enhancers of this risk as indicated in recent population-based studies (32–34). A more careful attention should be paid to this cause to address their consequences and better manage patients in order to mitigate their risks (35). The aim of this narrative review is to address new physiological and pathophysiological findings related to sodium and water disorders in chronic kidney disease (CKD) patients and to propose clinical action points to improve cardiac health in dialysis patients.

## 2 Sodium and Water Physiology: New Findings

### 2.1 Sodium Homeostasis: From Two to Three Compartment Model

In healthy humans, sodium and water homeostasis relies on a precise balance between sources (external dietary, internal metabolism) and losses (kidney, gut) in which kidney function and neuroendocrine factors play a major role. Traditionally, total body sodium (TBS) distributed in the extracellular volume (ECV) and bone, was thought to remain relatively constant over time and adjusted to intake changes. This fine regulation of TBS depends on kidney function and blood pressure as described in a kidney-centric model elaborated by Guyton et al (36). According to this view, sodium distributes in two compartments, circulating (volemia) and Interstitium, and ensures extracellular volume and hemodynamic homeostasis (36). In this setting, natremia reflects the effective plasma tonicity (natremia x 2) acting as the main driving force for water repartition within extra- and intracellular compartments. This two-compartment model depicts sodium in solution (i.e., osmotically active sodium), that controls blood pressure and hemodynamics to ensure adequate tissue perfusion. In the past decade, this conventional model has been challenged by new physiological findings that demonstrated the existence of a tissue sodium storage compartment, thus expanding the TBS concept into a three-compartment model (37–39). Initially, this model has been suspected from discrepancies in sodium mass balance studies conducted in healthy astronauts as part of the MARS 500 project (40). In conditions of strictly isolated and controlled conditions, the investigators observed that subjects submitted to precise diet salt intakes exhibited cyclic variations of TBS which were not translated in body weight or urinary sodium excretion changes but correlated with aldosterone and cortisol changes (40–43). This observation suggested that sodium accumulates in a third compartment without commensurate water retention. Later, this hypothesis was confirmed by means of sodium MRI imaging of (23)Na showing sodium stored in skin and muscle (42, 44). Tissue sodium concentration may be then quantified by (23)Na MRI (34, 44). From reference studies, tissue sodium is estimated between 10 and 40 mmol/l increasing with aging and under pathologic conditions (e.g., diabetes, hypertension, chronic kidney disease) (32–34). Further interventional studies confirmed that skin sodium storage is an active process that may be modulated according to sodium and water needs and conditions. Skin sodium is stored under the keratinocyte layer in a hypertonic environment that may regulate its own electrolytic microenvironment by means of sodium gradient and adjustment of lymph flux *via* angiogenic factors (45–48). Phagocytes sense hypertonic sodium accumulation and trigger tonicity-responsive enhancer binding proteins (Nuclear Factor of Activated T Cells 5, NFAT5) that stimulate in turn secretion and release of vascular endothelial growth factor C (VEGFC) (49). This mechanism has a dual action: first, it increases tissue sodium clearance *via* lymphatic flow; secondly, it acts on systemic blood pressure by modulating vascular tone *via* a stimulation (endothelial Nitric Oxide Synthase, eNOS expression. In addition, muscle sodium content tends to parallel skin behavior contributing to the overall tissue sodium storage with the same consequences. It has been suggested that tissue sodium content may contribute to blood pressure control independently from traditional neuroendocrine mechanisms *via* a skin immune-mediated mechanism (37). This interesting pathway of hypertension has been recently challenged by transgenic mouse models (50, 51). In these animal models, hypertension was induced by pathological losses of free water. Interestingly, hypertension was due to cutaneous vasoconstriction to limit epidermal water loss, and metabolic adaptation (muscle and protein catabolism) to enhance production of urea and organic osmolytes. This concept of pathogenesis of hypertension is antipodal to the classic view relying on salt retention (52). All these finding open new pathways for understanding better and managing hypertension resistant to traditional approaches (37).

In anuric chronic kidney disease dialysis patients, sodium and water balance rely mainly on renal replacement treatment schedule, dietary intake and likely on skin, gut, lungs and oxidative process. Conventional, short and intermittent hemodialysis treatment schedules create cyclic fluctuation alternating between a slow loading phase (interdialytic) and a fast-unloading phase (intradialytic), making fluid and volume management quite challenging (27, 29). As discussed earlier, fluid volume and pressure management expose patients to dialysis-induced systemic stress and morbidity. On one hand, chronic fluid overload is associated with mechanical and functional cardiac stress leading to structural changes contributing to cardiac remodeling (i.e., left ventricular hypertrophy, concentric or asymmetric, myocardial fibrosis, arrhythmias) and vascular consequences (atherosclerosis). On the other hand, acute fluid depletion secondary to ultrafiltration induces hypovolemia leading likely to intradialytic hypotension depending on patient’s hemodynamic response. In that setting, repetitive silent ischemic cardiac insults (i.e., myocardial stunning) may aggravate and accelerate damaging processes. Subsequently, further factors (i.e., arrhythmia, hypoxemia, ionic fluxes) may precipitate cardiac events and potentiate the effects of dialysis-induced systemic stress. Today, it is well recognized that maintenance conventional hemodialysis may act as a disease modifier likely contributing to end organ damage and cardiac burden (29). Aside management of volume and pressure, reflecting sodium osmotically active, a component that has been extensively studied, the role of tissue sodium accumulation and its management remain unexplored in maintenance hemodialysis patients (28, 53, 54). This new identified issue should be addressed more precisely in future clinical research (55). Indeed, it is speculated that restoration of tissue sodium homeostasis will be integrated in a more comprehensive management of sodium, water and blood pressure to improve cardiac health.

### 2.2. Pathophysiological Consequences of Sodium and Water Disorders in Advanced CKD and Dialysis Patients

Impairment of the sodium metabolism in patients with ESRD often results from longstanding pathologic processes that start early with kidney disease and aggravates steadily over time with progredient loss of kidney function. Sodium imbalance reflects the inability of the diseased kidney to handle daily sodium and water load.

Sodium accumulation in the extracellular space is the most common and diagnosable consequence of sodium imbalance in CKD patients. This refers to sodium in the extracellular fluid volume, the so called osmotically active sodium, which tends to increase thirst with subsequent expansion of the extracellular volume in the circulation and the interstitial space. Edema, hypervolemia, hypertension and congestive heart failure are among the most common manifestations of sodium accumulation and fluid overload translating into consequent cardiac and vascular damages. This pathophysiological dynamic has rendered chronic fluid overload with or without hypertension to be recognized as one of the main causes of morbidity in CKD patients. However, as summarized in recent reviews, next to sequelae on fluid and hemodynamics, sodium imbalance also associates with multiple end organ damages including cardiac remodeling (left ventricular hypertrophy), proarrhythmic condition, white matter brain damage, atherosclerotic lesion, protein energy wasting, inflammation and lung disorder with pulmonary hypertension (37, 55).

On the other hand, predialysis hyponatremia, reflecting hypotonicity and sodium-free water excess, is observed in 10 to 15% HD patients (56). Hyponatremia is a strong marker of poor outcomes (9, 13, 56, 57). Recent studies have shown that hyponatremia is in fact a mixed fluid disorder, consisting of extracellular fluid excess and water compartment repartition imbalance, reflecting an underlying illness such as congestive heart failure, liver disease or inflammatory protein energy malnutrition (10, 13, 15, 58). Interestingly, recent observational studies have shown that clinical outcomes might be improved by intensifying fluid and sodium depletion [negative dialysate-to-plasma (d-p) Na gradient] rather than correcting plasma sodium concentrations while loading patient by applying a positive (d-p) Na gradient (9).

Sodium accumulation in tissue, as discussed in the previous paragraph, also associates with chronic kidney disease progression. As documented through functional (23)Na MRI imaging, skin and muscle sodium content increase steadily over time as kidney function deteriorates (32). Interestingly, several metabolic consequences of salt tissue accumulation and organ damage have been identified as an independent component of its mechanical action (55). However, this is a new field of research with fast growing and fascinating findings in which more must be discovered. Few examples will be used to illustrate this new pathophysiologic link. Left ventricular hypertrophy has been shown to be positively correlated with skin sodium content, almost independently from blood pressure level, in a prospective study conducted in advanced non-dialysis CKD patients (53). In HD patients, pulse wave velocity (PWV) changes may likely reflect vascular sodium content changes and endothelial function improvement as suggested by acute changes in PWV following sodium depletion by dialysis (59–61). Insulin resistance, assessed by means of euglycemic clamps, has been found to be inversely correlated to skin sodium content in HD patients, suggesting that tissue sodium interacts with insulin pathways independent of uremic toxin levels (62). Salt loading and tissue storage activate an adaptive regulatory network mechanism in the muscle that enables reprioritization of local energy metabolism and induces muscle wasting in healthy subjects (63). In a recent clinical case report, refractory pruritus has been linked to a massive skin sodium accumulation in a HD patient and improved after large sodium depletion through expanded hemodialysis (64). This latter observation led investigators to speculate on a possible link between skin salt accumulation and local immune mechanisms activating keratinocytes (64).

## 3 Implications of Intermittent Treatment of Maintenance Hemodialysis: Unphysiological Profile Induced by Intermittent Therapy

The unphysiological profile of intermittent HD is recognized as a leading cause of dialysis intolerance and multiorgan morbidity (65, 66). This phenomenon is worsened by short or very short dialysis treatment schedules. Intermittent HD generates periodic and cyclic changes in volume and blood pressure, osmotic shifts, and fluctuations of waste products and electrolytes (67). Cyclic profiles are contrasting with closely regulated and relatively stable conditions of the internal milieu in healthy or even non dialysis CKD patients.

The HD cyclic phenomenon may refer to a tide phenomenon with two phases of loading (interdialytic) and unloading (intradialytic) as described in more details below (27, 35, 67).

During the interdialytic period, anuric HD patients tend to accumulate sodium and fluid according to fluid and dietary intake, leading to chronic fluid overload. In this condition, fluid overload has two consequences: the first is marked by weight gain and progressive increase of systemic arterial pressure and pulmonary arterial pressure with cardiac stretching during the interdialytic phase; the second reflects fluid accumulation and translates into cardiac stretching and structural cardiac remodeling (68).

During the intradialytic period, sodium and fluid are removed mainly through ultrafiltration (intradialytic weight loss) and negative dialysate-to-plasma sodium gradient. Volume depletion leads to hypovolemia that triggers adaptative hemodynamic response to preserve arterial pressure. To face hypovolemia and cardiac stroke reduction, hemodynamic stability (blood pressure and tissue perfusion) tends to be preserved by increasing vascular tone, mainly through vasoconstriction of alpha-adrenoceptor territories, and increase vascular refilling including venous return. Hemodynamic response and full adaptive response may be limited by critical hypovolemia (ultrafiltration to refilling imbalance) or cardiac impairment (diastolic and systolic dysfunction, arrhythmia, heart failure) or vascular refilling capacity (hypoalbuminemia, capillary albumin leakage, inflammation). Recent functional dialytic imaging studies have shown that reductions in myocardial perfusion and contractility (myocardial stunning) are directly linked to ultrafiltration rate (69–72). In addition, it has been shown also that this phenomenon starts very early during HD session even before ultrafiltration has reach a significant level that may reduce volemia (73, 74). Several observational studies have documented a strong association between mortality and high ultrafiltration rate or volume changes, drop in blood pressure, and end-organ ischemic insult (75, 76). Indeed, hemodynamic response to hemodialysis is more complex than a simple reaction to hypovolemia, since it includes other factors such as vascular refilling capacity, bioincompatibility reactions, thermal balance, electrolyte fluxes, nutrient losses and individual patient’s characteristics (cardiac reserve, neurohormonal stress responses) (28, 29). Interesting, this response may be mitigated by various factors (e.g., age, gender, comorbidity, autonomous neuropathy, and medication) explaining also individual or temporal variations in hemodynamic response.

Whatever the exact pathophysiologic consequences of this phenomenon, volemic changes (hyper- and hypo-volemia) provoke rapid alternating cycles of cardiac loading and unloading but still maintaining overtime an abnormal high pulmonary pressure level (68, 77). Such cycling phenomenon is responsible for repetitive and chronic myocardial stretching, a mechanism that has been recognized by cardiologists as the main mechanism of inflammatory mediator release (78, 79). This mechanism contributes to cardiac remodeling and further fibrosis, a proarrhythmogenic condition (79).

## 4 Actions to Mitigate CV Risk Associated With Sodium, Water, Volume and Hemodynamic Management

Adequate management of sodium, water, volume and hemodynamic control of hemodialysis patients relies on a stepwise approach (28, 67, 80): the first entails assessment and monitoring of fluid status and relies on clinical judgement supported by specific tools; the second consists in acting on correcting fluid imbalance mainly through dialysis prescription but also on diet and thirst guidance; the third consist in fine-tuning treatment prescription to patient response and tolerance.

### 4.1. Monitoring Sodium, Water and Fluid Status

**Figure 1:**
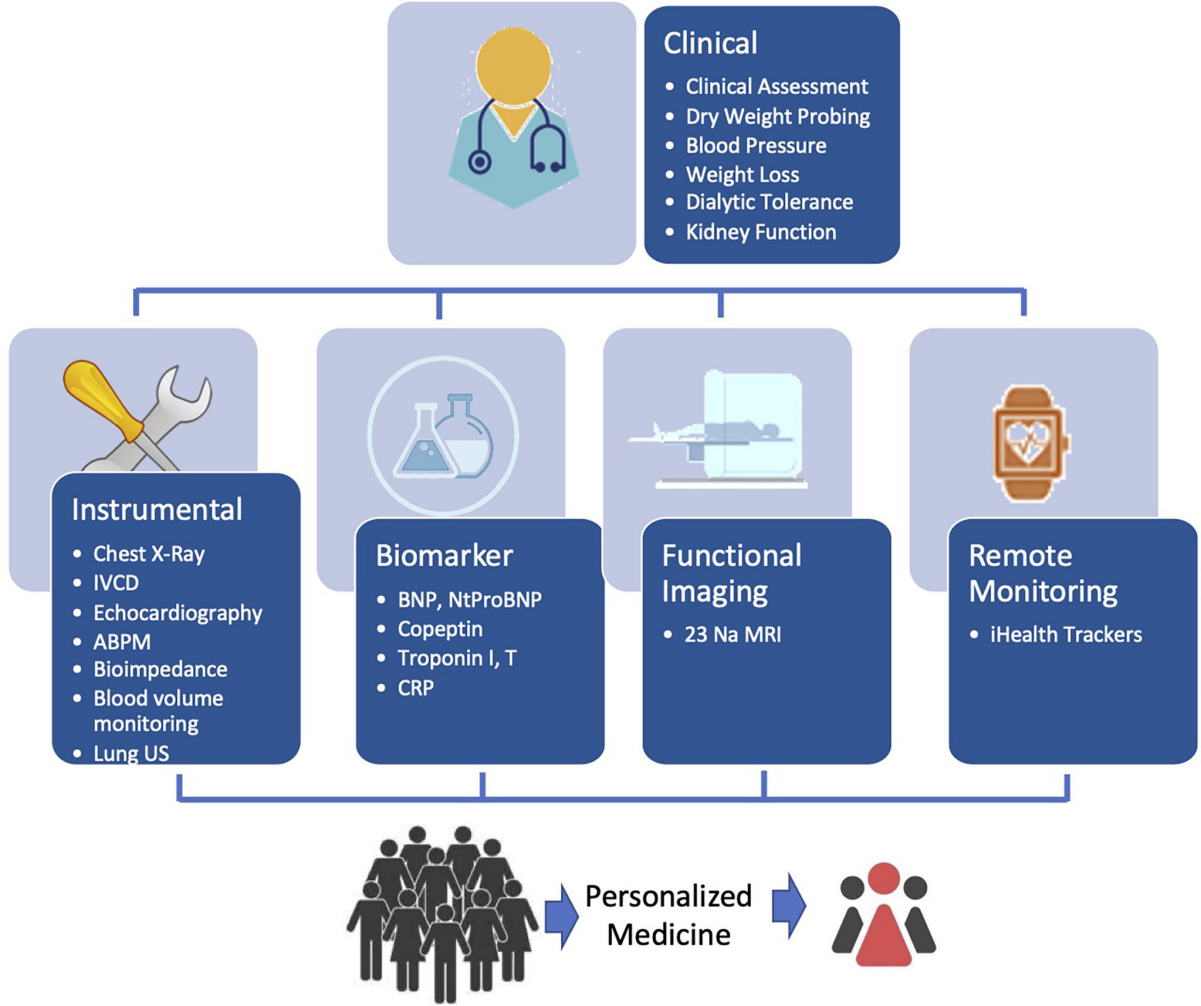
Schematic diagram for optimizing and integrating tools for personalized guidance of fluid management in HD patients.

Sodium and fluid balance assessment and monitoring in HD patients is not an easy task (81). However, this is the first step from a clinician’s perspective to ensure a better and more precise salt and volume management. Patient monitoring relies on a clinical judgment supported by several tools depending on the complexity of the case as depicted in **Figure 1**.

#### 4.1.1 Clinical assessment

Clinical management is currently summarized in the dry weight probing approach (82–84). The concept of dry weight was introduced by Scribner and colleagues in the 1960s, as the main pathway to control fluid overload and blood pressure in HD patients. Dry weight probing involves a stepwise reduction of post dialysis weight over time to achieve an adequate control of systemic blood pressure, disappearance of fluid overload symptoms and prevention of intra- or peri-dialytic hypotension. This clinically-oriented approach has been shown to be associated with indisputable value in clinical nephrology (85). As just confirmed recently in interventional studies this clinical approach allows for good control of blood pressure and to improve long-term patient outcomes (86). However, sensitivity and specificity of clinical assessment in detecting fluid imbalance as set by clinically determined ‘dry weight’ is challenged when compared to instrumental tools such as bioimpedance (87, 88). On the other hand, excessive or too fast fluid depletion has led to some concerns as being associated with higher risks including cardiac stunning or severe cardiovascular events (88–90). This is now being recognized as being part of dialysis-induced systemic stress. Volemia management remains a critical concern in HD patients that has been recently highlighted by the KDIGO controversy conference (91). Therefore, if dry weight policy remains still valid and necessary from a clinician perspective, it is not sufficient to ensure optimal sodium volume and pressure management in dialysis patients. Further tools are required to support clinical decision making on a daily practice.

#### 4.1.2 Instrumental Tools

Non-invasive technology-based tools have been shown helpful to assess volemia, fluid status, or hemodynamic indicators (67).

Inferior vena cava diameter (IVCD) and collapsibility index have been proposed to monitor intravascular volume and right atrial pressure or central venous pressure changes in dialysis patients with positive outcomes (92). However, the practical difficulty in implementing these methods, factors affecting reading and the poor predictive value on blood pressure response in probing dry weight have precluded its generalizability in chronic patients setting (93).

Relative blood volume change (RBV) and refilling rate capacity during dialysis assessed by online blood volume sensor embedded in HD machines has been also proposed to guide fluid management (94, 95). In expert hands, this tool may provide at bed side useful information on individual patient intravascular volume status to handle hemodynamic guidance (96). Blood volume monitoring may be used to better characterize patient’s critical volemia beyond which occurrence of severe intradialytic hypotension is likely to occur (97). Absolute blood volume measurement, based on non-invasive measurement either by dilution or online calculation, has been proposed recently for a better assessment of this crucial parameter (98). To date, only one limited study has explored the clinical benefits of monitoring precisely this parameter. An observational study in 842 hemodialysis patients has shown the existence of certain “favorable” RBV ranges at specific timepoints during dialysis that are associated with improved patient survival (99). These RBV ranges have been recently integrated as control targets in an automatic UFR feedback concept (100).

Bioimpedance has been proposed over the last few years as a more precise way to assess fluid status in dialysis patients (101, 102). Several approaches (segmental versus total body, single versus multifrequency) using various devices and algorithms have been developed with interesting results (103). In a systematic review, multifrequency bioimpedance spectroscopy (BIS) analysis [National Institute for Health and Care Excellence,(NICE, UK)] was recognized as the most precise and reliable tool in a clinical setting for guiding fluid management in dialysis patients (104). In addition, extensive use of BIS in clinical studies has generated substantial evidences showing that BIS was able to detect subtle fluid volume variation and to support clinical decision making in terms of dry weight reduction (105). Although some studies showed positive effects of fluid management based bioimpedance on clinical endpoints, there are most based on observational data while controlled interventional studies are still lacking for generating stronger evidences.

Echocardiography is a reliable tool in expert hands to monitor cardiac impact of fluid depletion both on functional and morphological aspects (106–108). It has been shown that several cardiac key parameters such as ejection fraction, left ventricular mass, left ventricular end diastolic volume, peak strain, aortic distensibility or pulmonary arterial pressure or right atrial volume have associations to chronic fluid overload and are useful or be regularly assessed in dialysis patients. However, while their use for dry weight determination has been proposed, most agree on their limitations for that particular purpose.

Regional chest bioimpedance cardiographic device (NICaS), a non-invasive device, has been recently introduced to assess patient’s hemodynamic response to ultrafiltration (109). The device relies on skin electrodes and sophisticated proprietary algorithms integrating ECG and blood pressure parameters and claims the remarkably ability to determine stroke volume, cardiac index and power and total peripheral vascular resistance. Based on this set of parameters, it is possible to identify different profiles of hemodynamic response in terms of peripheral vascular resistance and cardiac function (110). In this pilot study, the authors identified that hemodynamic response to fluid depletion and/or hypovolemia (ultrafiltration) may differ substantially according to patient profile. Based on NICas, three groups of patients were identified: firstly, predominant decrease of cardiac power index (reduction of blood pressure and cardiac output reflecting preload reduction); secondly, predominant decrease of peripheral vascular resistance (autonomous dysfunction); thirdly, both decrease of cardiac power index and total peripheral vascular resistance (combined phenomenon). Such assessments in dialysis patients may facilitate interpretation of hemodynamic response (cardiac dysfunction, insufficient vascular refilling capacity, autonomous dysfunction) and then help nephrologists to optimize fluid removal preventing intradialytic hypotension.

Lung ultrasound has been proposed more recently to track silent fluid accumulation in the lung Interstitium (extravascular edema) reflecting both volume overload and cardiac dysfuntion (111, 112). Interlobular septa thickening due to water accumulation reflects US beam and generates visible B line bundles (comet- like tails). A simple counting of these B lines provides an estimate of lung water excess and may support clinical decision-making in terms of dry weight probing (113, 114). The approach has been shown beneficial to reduce fluid overload and blood pressure levels in a recent controlled trial investigating the management of dialysis patients with resistant hypertension (112). A recent interventional trial has explored the clinical interest of using predialysis lung ultrasound scan (Lung Ultrasound Study, LUST study) including 183 patients in the active arm versus 180 in the control arm, to titrate ultrafiltration during dialysis. Lung congestion was significantly more frequently relieved in the active (78%) than in the control (56%) arm. However, risk for all-cause and cardiovascular hospitalization and the changes of left ventricular mass and function did not differ among the two groups suggesting that better volume control is not sufficient per se to reduce cardiac burden in dialysis patients. In addition, a *post-hoc* analysis for recurrent episodes of decompensated heart failure (HR 0.37) and cardiovascular events (HR 0.63) showed a significant risk reduction in the active arm. In hemodialysis patients with high cardiovascular risk, fluid management guided by lung ultrasound may help to reduce lung congestion more effectively than usual clinical care (115).

#### 4.1.3 Cardiac Biomarkers

Cardiac biomarkers have been extensively explored in hemodialysis patients to disentangle fluid status and cardiac function or remodeling (116). Atrial natriuretic peptides (ANP, BNP, and NT-proBNP) are the most commonly used ones for assessing fluid overload (117–119). More recently, copeptin (a vasopressin precursor) has been recently introduced to assess fluid depletion (120). Cardiovascular biomarkers reflecting cardiac or endothelium injury are also of interest to set a more precise and personalized fluid management approach. Sensitive troponin from the family of troponin molecules (troponin I and T) have been used to detect or to prevent critical cardiac injury in response to fluid depletion (121, 122). Several endothelial biomarkers (e.g., ADMA, FG23, ROS, NO pathways) or inflammatory mediators (CRP, IL1, IL6) or oxidative stress markers have further been proposed, either isolated or combined, to assess cardiac and vascular risk as part of the fluid management strategy with promising results (123, 124). As shown in the few prospective cohort studies, Brain Natriuretic Peptides (BNP) or their surrogates were used successfully to better guide fluid management in incident dialysis patients with past cardiac history or when hospitalized for cardiac decompensation (118, 119, 125).

#### 4.1.4 Functional Imaging Tools

Quantification of sodium accumulated in the tissue (skin and muscle) using sodium (23)MRIs in dialysis patients have become the focus of several investigations to assess tissue sodium (32, 44). As outlined before, accumulation of sodium in the tissue may contribute to systemic toxicity *via* local or remote tissue organ damage. Consequences such as left ventricular hypertrophy and vascular stiffness positively associate with the amount of tissue sodium stored and may ultimately increase the risk of cardiac failure. In addition, sodium tissue accumulation contributes to metabolic and inflammatory disorders that enhance cardiovascular risk. In recent study assessing tissue sodium (skin, muscle) by (23)Na MRI in dialysis patients, it has been shown that tissue sodium and water were mobilizable by hemodialysis (54). A reduction of almost 50% of tissue sodium concentration (skin and muscle) was observed contributing to the net salt mass recovered from a direct dialysate quantification. However, tissue Na removal was apparently not linked to dialysate-plasma Na gradient. Sodium (23)MRI remains clinical research tool with restricted access due to its complexity. However, it is envisioned that dedicated segmental sodium MRI device will be available in the near future (126).

#### 4.1.5 Remote Monitoring Tools

New remote technology, so called ihealth trackers, offer convenient and interesting tools for monitoring in a fully automated and non-obstructive mode, HD patients during the interdialytic period (127). Long-term remote monitoring of vital signs, blood pressure, heart rate, respiration rate, physical activity appears to be a valuable approach for assessing high risk dialysis patients knowing that sudden cardiac death occurs mostly during interdialytic phases. In addition, home based connected devices such as electronic weighting scale with integrated bioimpedance may facilitate monitoring of fluid volume gain in the future (128). Best use of these tools may help clinicians to identify earlier clinical conditions of fluid imbalance or cardiac conditions in order to act on, before they reach critical stage.

### 4.2. Acting on Sodium, Water and Fluid Management

#### 4.2.1 Dry Weight Probing Approach: Clinical Management

**Figure 2:**
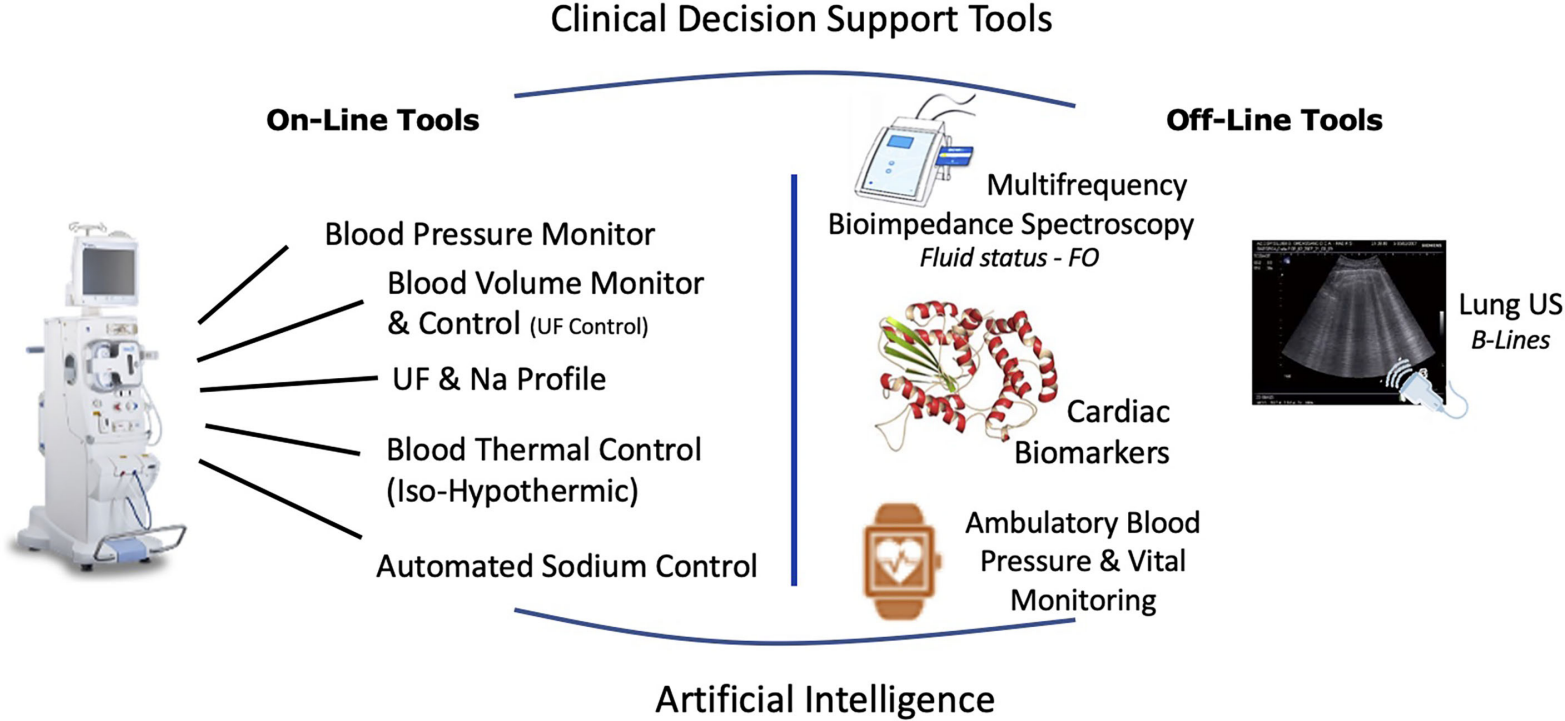
Integrated approach to reduce intradialytic hypotension and to reduce dialysis induced systemic stress relying on current available integrated tools on HD machines and potentially supported by specific cardiac intervention.

Dry weight clinical management must be conceived as the overarching workflow aiming to ensure optimal fluid and hemodynamic management in dialysis patients (83, 129). This process relies on four main components that include diet, hemodialysis prescription, residual kidney function and adjunctive medication. Success of dry weight achievement is then evaluated at short term on objective key parameter indicators (i.e., lack of clinical symptoms, optimal blood pressure, euvolemia, normal BNP levels) and patient well-being. This is visually outlined in **Figure 2**.

#### 4.2.2. Diet Counseling: Reduce Salt Intake

Reduce dietary salt intake is associated with clinical benefits, better cardiac outcomes in chronic kidney disease and hemodialysis patients. Adherence to a low-sodium diet is challenging but well established as crucial element for treatment success (130). Salt dietary counselling should be better implemented through renal dietitian educational support on an individual basis accounting for lifestyle and diet habits. It is not our intent to review salt dietary recommendations, we refer interested readers to recent reviews (131, 132). In brief, it is currently recommended to restrict salt (sodium chloride) diet intake to about less than 5 g (85 mmol) per day that it equivalent to 10 g (170 mmol) between two HD session (91). Apart from cardiac health, salt diet restriction has additional benefits in HD patients since it reduces thirst and interdialytic weight gain facilitating HD management.

#### 4.2.3. Treatment Time and Frequency: Reduce Ultrafiltration Rate While Increasing Net Ultrafiltration

Reducing ultrafiltration rate is obviously the most logical approach to reduce cardiac morbidity in HD patients (71, 133, 134). Aggressive management of sodium and fluid excess to restore fluid homeostasis either by applying high ultrafiltration rate and furthermore associating hypertonic dialysis (high dialysate-to-plasma sodium concentration gradient) to facilitate refilling rate has been associated with increased risk of mortality. The optimal and rational approach to improve excess fluid volume is to increase, either HD frequency and/or dialysis time or to have additional isolated ultrafiltration sessions. Daily or nocturnal dialysis treatment schedule have been proved to be associated with significant improvement in fluid and hemodynamic management in HD patients (135–138). Nevertheless, these approaches may not always be accepted by patients or applicable by care providers for organizational or economic reasons.

#### 4.2.4. Blood Volume Control: Prioritize Blood Volume Preservation

Modulating a patient’s hemodynamic response through various tools embedded in HD machines is an appealing approach to maintain hemodynamic stability (94). Monitoring blood volume changes during HD session is useful to identify critical volemia (i.e., intradialytic hypotension risk), to estimate remaining fluid in the Interstitium, or to quantify vascular refilling capacity, but it is not sufficient to optimize hemodynamic response (139, 140). Additional feedback-controlled loop algorithm set on critical volemia threshold and acting on ultrafiltration is better suited to provide a precise preservation of effective volemia (ultrafiltration control) (141). This tool could be coupled to other options such as sodium management and profile (142, 143). Ultrafiltration control tool improves patient’s hemodynamic tolerance, reduces intradialytic hypotension and cardiac stunning risks (144). In brief, ultrafiltration control tends to reduce cardiac insult but its long-term clinical benefits remain to be proven.

#### 4.2.5. Thermal Balance: Favor Iso or Hypothermic Dialysis Condition

Adjusting dialysis thermal balance to preserve peripheral vascular resistance and cardiac output is also a simple strategy to improve hemodynamic tolerance and reduce organ damage that has been proven clinically effective including systematic literature review (142, 145, 146). In brief, the main objective is to deliver isothermic (patient’s neutral thermal balance) or hypothermic dialysis (patient’s negative thermal balance), to prevent thermal gain during a dialysis session which is associated with an inappropriate hemodynamic response (vasodilation, tachycardia, fall of ejection fraction) (147). Hypo-or isothermic HD could be manually achieved by setting dialysate temperature 0.5-1°C below predialysis patient’s core temperature. Automated thermal control of dialysis sessions requires the use of an online blood temperature monitor that can control more precisely thermal balance of patients to a preset target. Both approaches tend to reduce hypotension incidence, hemodynamic stress and organ insult as shown in recent studies (145, 146).

#### 4.2.6. Sodium and Water Control: Prioritize Isonatremic HD and Sodium Mass Removal

Optimizing sodium and water imbalance by means of hemodialysis is crucial to restore fluid and tonicity homeostasis (148, 149). This process relies on convective and diffusive sodium flux times treatment time. The sum of convective and diffusive fluxes determines total salt mass removed per session (55, 150). Convective sodium flux is dragged through ultrafiltration (weight loss), while diffusive sodium flux is driven by a dialysate-plasma sodium gradient. In that setting, dialysate sodium concentration plays a particular role in sodium management since it acts both on sodium mass removal and on plasma tonicity changes. Dialysate-plasma sodium gradient prescription rather than dialysate sodium concentration alone should be considered for personalizing dialysis prescription (150). Indeed, there is no medical rationale today to prescribe dialysate sodium on a fixed concentration basis except to facilitate facility practice. In all cases, predialysis plasma sodium concentration (or mean value) should be used as reference value for choosing dialysate sodium prescription. Manual dialysate sodium alignment to predialysis plasma sodium concentration may be then reconsidered on a periodically basis according to results (151, 152). An innovative approach for dialysate sodium prescription has been proposed recently relying on an automated sodium balancing module that has capacity to align dialysate sodium concentration to plasma sodium according to physician prescription (153–156). Based on this new tool, care givers have the opportunity to customize dialysate sodium prescription according to patient needs (sodium mass and tonicity adjustment) in an easy and timely appropriate manner without the cumbersome task of laboratory testing. Accordingly, one may identify three prescription options: positive gradient (or hypertonic dialysis), neutral gradient (or isonatremic dialysis) or negative gradient (or hypotonic dialysis). For safety reason, positive gradient will be preferably ranging between +1 to +5 mmol/l; negative gradient will be ranging between -1 and -5 mmol/l. Isonatremic dialysis will be then ranging between -1 and +1 mmol/l. Isonatremic dialysis may represent default basic prescription for the majority of patients. Hypotonic dialysis may be favored in patients with resistant or paradoxical hypertension, fluid overload or tissue sodium excess to enhance sodium depletion. Hypertonic dialysis may be indicated in hypotensive prone or hypovolemic patients in order to improve hemodynamic tolerance. In addition, it is expected that continuous fine tuning of dialysate sodium alignment on plasma sodium concentration may facilitate sodium mobilization from tissue storage addressing more adequately total body sodium homeostasis (157). Further focused clinical studies are clearly required to better identify potential benefits or risks associated with these personalized prescriptions. Alternatively, the use of sodium control module to deliver isotonic dialysis is likely expected to reduce thirst by preserving the patient’s tonicity set point (158). Potential benefits of isotonic or zero-diffusive dialysis are currently explored in various prospective studies (159).

### 4.3. Predictive Medicine, Advanced Analytic and Artificial Intelligence

Big data and artificial intelligence have already been successfully applied at the point of care to support physicians in the decision-making process. Availability of accurate, longitudinal, data set is a key factor for the development of reproducible predictive algorithms. In that setting, artificial intelligence relying on machine learning, neuronal network and deep learning, can be used to predict on an individualized, session-based, patient hemodynamic response (intradialytic hypotension) to dialysis-related prescriptions (ultrafiltration, dialysate sodium, treatment time, dialysis modality) on multiple relevant hemodynamic and dialysis adequacy parameters (160). Based on this information, clinician can choose on time at the point of care the best dialytic strategy to reduce hemodynamic stress for a given patient. Value of this approach deserves further clinical trials.

### 4.4. Adjunctive Actions: Cardiac Management: Medications, Synchronization

Additional specific actions may be further required in cardiac compromised patients or to satisfy specific patient’s needs (161). It is not our intent to make an in-depth review of cardiac interventions to improve cardiac health. However, few examples may be presented to illustrate our purpose. Renin angiotensin blockers or calcium channel blockers may be indicated in case of refractory hypertension (162). Betablockers, renin angiotensin inhibiting agents or mineralocorticoid receptor antagonists may be also indicated in ischemic cardiac disease or in obstructive or diastolic cardiac failure (163–165). In that setting, the right medication (preferably non-dialysable) with the appropriate dosing is needed to prevent clearing and loss during dialysis. Coronary angioplasty, or coronary bypass as well as valve replacement should be envisaged as needed (166). Cardiac resynchronization relying on pacemaker implantation may be indicated in case of severe cardiac dysfunction. Implantable defibrillator may be indicated in case of severe and repetitive arrhythmia associated with risk of sudden cardiac risk (167). Cardioversion may be indicated in selected cases of atrial fibrillation resistant to medication with interesting results (168).

In brief, indications of these medications and/or cardiac intervention should remain in the hand of cardiologists. At this stage, our point is to emphasize the fact that sodium, water and fluid imbalance should be the first line of action in HD patients. Medications and cardiac interventions are likely to be useful but should remain as second line of action and nevertheless used in combination with optimized sodium and water management.

## 5 Perspective for Future Improvement

**Figure 3:**
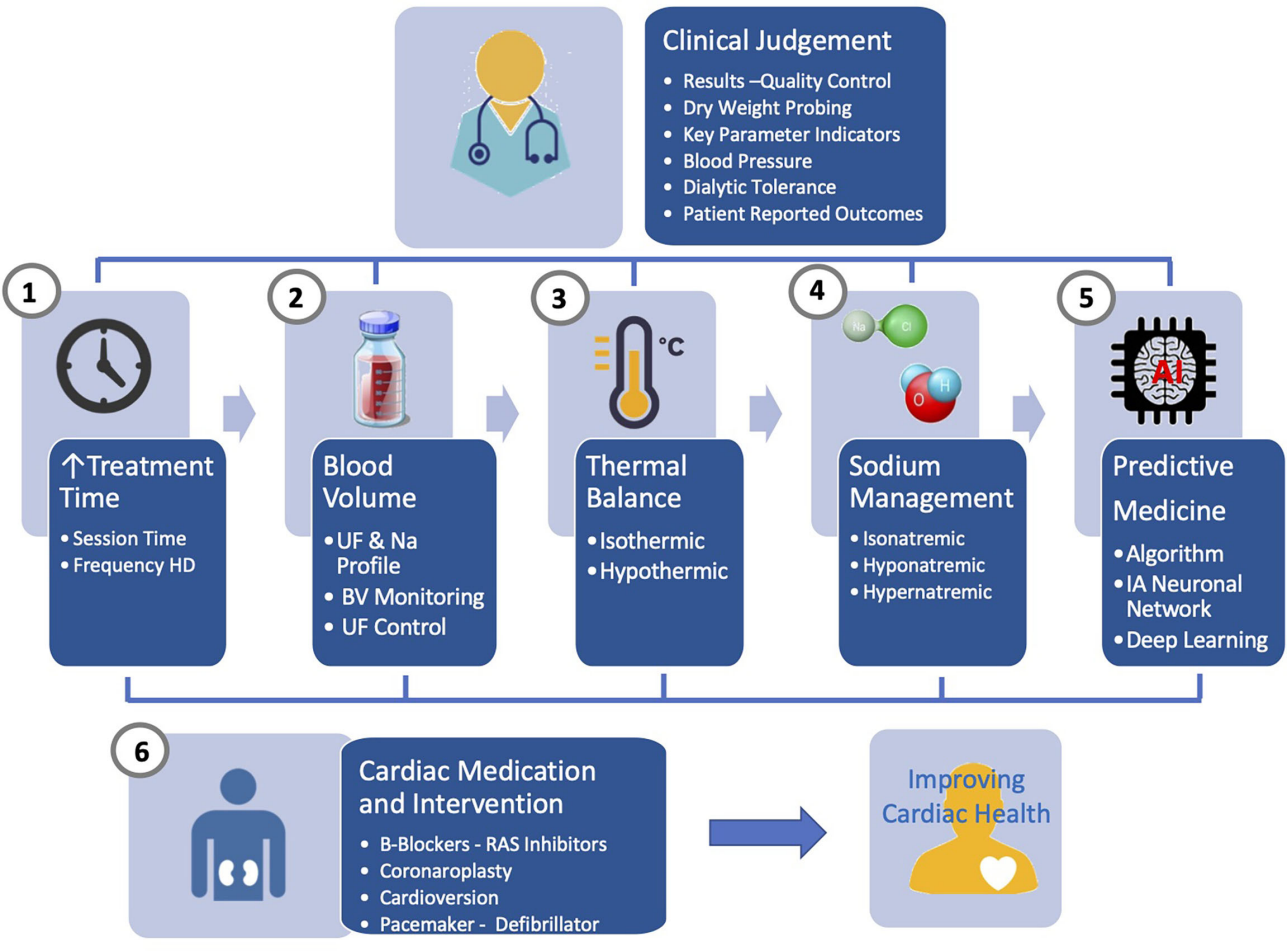
Advanced management of sodium, fluid and blood pressure in hemodialysis patients integrating currently available online and offline tools under the clinical supervision and supported by artificial intelligence.

A comprehensive and integrated approach is needed to improve further cardiac outcome in HD patients. From clinical research, it is obvious that none of the tool described earlier used alone has the capacity to address issues raised by sodium, water and fluid imbalance. Future research should address this challenge by associating different levels of action as briefly schematized in **Figure 3**. firstly, relying on online tools embedded in HD machines (i.e., ultrafiltration-controlled volume, thermal balance, sodium control module) and secondly, on offline tools (i.e., bioimpedance, lung US, ihealth trackers) for fluid and pressure status monitoring. In that perspective, biosensors use has to be orchestrated and integrated into specific algorithms and feedback loops control as part of smart HD machine providing immediate support to care givers; secondly, using offline tools (bioimpedance, biomarkers) feeding network data system, benefiting from advanced analytics and artificial intelligence, supporting in almost real-time clinical decision making; thirdly, optimized functioning of these tools will rely on large web based networking system (big data, cloud computing) that can integrate data from all sources of information and propose clinical guidance to care giver.

## 6 Conclusion

As delineated in this comprehensive essay, new findings related to sodium homeostasis and pathophysiologic links require a new vision for sodium, fluid and pressure management in dialysis dependent chronic kidney disease patients. The traditional dry weight probing approach that has prevailed for many years must be reviewed in the light of these new findings and enriched by availability of new tools for monitoring and handling sodium and water imbalance. It is time to come back to sodium and water imbalance as cause root of the problem and not to act on their consequences (fluid overload, hypertension) or organ damage (cardiac, atherosclerosis, brain damages). We know the problem and have the tools to assess and manage in a more precise way sodium and fluid disorders in hemodialysis patients. We strongly call for a sodium first approach to reduce disease burden and improve cardiac health in chronic kidney disease patients.

## Author Contributions

All authors listed have made a substantial, direct, and intellectual contribution to the work, and approved it for publication.

## Conflict of Interest

Authors AM and BC were employed by company Fresenius Medical Care (FMC).

The remaining authors declare that the research was conducted in the absence of any commercial or financial relationships that could be construed as a potential conflict of interest.

## Publisher’s Note

All claims expressed in this article are solely those of the authors and do not necessarily represent those of their affiliated organizations, or those of the publisher, the editors and the reviewers. Any product that may be evaluated in this article, or claim that may be made by its manufacturer, is not guaranteed or endorsed by the publisher.
